# A New Approach to the Reduction of Alcohol Content in Red Wines: The Use of High-Power Ultrasounds

**DOI:** 10.3390/foods9060726

**Published:** 2020-06-02

**Authors:** María Pilar Martínez-Pérez, Ana Belén Bautista-Ortín, Paula Pérez-Porras, Ricardo Jurado, Encarna Gómez-Plaza

**Affiliations:** 1 Department of Food Science and Technology, Faculty of Veterinary Sciences, University of Murcia, Campus de Espinardo, 30100 Murcia, Spain; mpilar.martinez2@um.es (M.P.M.-P.); anabel@um.es (A.B.B.-O.); paula.perez2@um.es (P.P.-P.); 2 Agrovin, S.A. Av. De los Vinos s/n, Alcázar de San Juan, 13600 Ciudad Real, Spain; rjurado@agrovin.com

**Keywords:** ultrasounds, phenolic compounds, color, aroma, sensory analysis, alcohol content, wine

## Abstract

Background: To obtain wines with a lower percentage of alcohol, the simplest approach would be an earlier harvest of the grapes. However, this has implications for the wine composition and quality, due to the lack of phenolic maturity that these grapes may present. A technological innovation that could help in this situation could be the use of ultrasound in wineries. Methods: Grapes were harvested with two different ripening levels (25.4 °Brix and 29 °Brix), transported to the winery, and vinified. Also, a large-scale high-power ultrasound system was used to treat part of the less mature grapes just after crushing. These grapes were also vinified. The three different vinifications were skin-macerated for 7 days. The wine aroma compounds and physicochemical, chromatic, and sensory characteristics were analyzed at the time of bottling. Results: The wine made with the ultrasound-treated grapes showed very similar characteristics to the wine made with the more mature grapes, especially regarding total phenol and tannin content, but with an alcohol content 15% lower than the latter. Conclusions: The results indicate that this technology could be applied to grapes to favor the extraction of grape phenolic compounds, even when grape phenolic maturity is not complete, allowing the production of quality wines with a reduced alcohol content.

## 1. Introduction

Color is one of the most important quality attributes in red wine. It depends on the phenolic composition of the wine (a composition that does not only affects wine color, but also body and mouthfeel), and therefore, is closely bound to the grape phenolic composition.

Grape phenolic compounds are mainly located in the skin (anthocyanins and tannins) and seeds (tannins), and are extracted to must during the crushing and maceration period. Although some enological techniques may help to extract these compounds [1], this extraction can be seriously limited by the cell walls of the cells where these compounds are located, which form a barrier to their extraction [2]. If these cell walls are not easily broken down, the extraction of the phenolic compounds will be limited. From these observations, the concept of phenolic maturity has appeared at the grape stage, where the skin phenolic content is not only high, but also easily extracted, and seed tannin extraction is reduced due to a lignification of the seed [3]. When grapes are phenolically immature, the skin phenolic compounds are not easily extracted, even when present at high concentration, yet high concentrations of the astringent seed tannins can be present [4,5]. This situation changes when phenolic maturity is reached, cell walls are easily degraded, and phenolic compounds are extracted [2]. 

In optimal situations, and when a variety is well adapted to a certain area, technological maturity (the optimal level of sugar content in the grapes for a certain type of wine) and phenolic and aromatic maturity (when grapes have lost vegetal and herbaceous aromas and fruity aromas are expressed) are reached at the same time. However, climate change is exerting a large influence on vine phenology and grape composition [6], and among the most important climate change-related effects is a modification in vine phenology [7]. If vine phenology is moved to earlier dates, due to global warming, this can lead to an asynchronous development of grape composition, with the sugar accumulation being faster than the phenolic compounds synthesis. Consequently, a delay in harvesting in order to allow grapes to reach an optimal aromatic and phenolic maturity may lead to higher berry sugar levels than desired, and therefore higher alcohol content in wines [8].

The issue of high-alcohol wines is a big concern for winemakers, since it has potential implications for wine quality. Ethanol is sensorially important to wine, and is indispensable for the stability, aging, and organoleptic properties of wine [9]—and therefore, wine style. However, a high ethanol concentration may present some technological problems: it can be toxic for yeast cells, and as a result, lead to arrested or sluggish fermentation, as stated by Henderson and Block [10], and as stated by Boulton et al. [11], it could inhibit the malolactic fermentation. From a sensory point of view, it can influence our perceptions of astringency, sourness, flavor, and aroma, as recorded by some authors [12,13,14], and wines can be perceived as hotter on the palate [15]. Lastly but not least importantly, high alcohol content has negative effect on human health and can be more expensive, since they are taxed at higher rates in many countries [16].

Given all these issues, winemakers are really seeking different possibilities for obtaining high quality wines with a reduced alcohol content. Different approaches to reduce alcohol levels in wines have been proposed at all stages of the winemaking process, from the addition of unripe grape juice to finished wines [17,18], to the use of yeasts that have a low production of alcohol [19,20], or the use of technologies for partial dealcoholizing processes [21,22].

It could be, though, that one the easier solutions for wine alcohol reduction would be harvesting grapes with lower sugar content—if we can solve the problem of the low phenolic maturity that these grapes could present at this stage, and therefore, the difficulties in obtaining highly colored wines. Some enological techniques are focused in this issue, such as the use of maceration enzymes or prefermentative maceration techniques, as reviewed by Sacchi et al. [1], as well as novel technologies that could be used to solve this problem.

Among the novel technologies, high-power ultrasounds (HPUs) could be an interesting approach. This is a technology that has already been approved by the International Organization of Vine and Wine (OIV) in 2019 for its use in wineries. HPUs are generally comprised of frequencies between 20–40 kHz, with an energy level high enough to produce acoustic cavitation. This effect consists in the formation of tiny bubbles that grow until they reach a critical size that causes their implosion. During the implosion, remarkably high temperatures (circa 5000 K) and pressures (circa 2000 atm) are reached [23]. When this implosion occurs near a cell, the resulting forces can break the cell walls, leading to two potential results: in plant cells, it allows the diffusion of the compound located inside them [21], and in the case of a microorganism cell, it may lead to death of the organism itself [24].

Therefore, in enology HPUs could be used to

(a) Improve the extraction of phenolic and aroma compounds from grapes [25,26,27,28];
(b) Reduce the use of SO_2_ by reducing microbial counts. In this way, Gracin et al. [29] found that high power ultrasounds applied in continuous flow showed satisfactory reduction of *Brettanomyces* yeasts (89.1–99.7%) and lactic acid bacteria (71.8–99.3%), and Santos et al. [30] review the possibility of using ultrasounds in several stages of winemaking for wine conservation;
(c) Age wines on lees: Kulkarni et al. [31] used non-*Saccharomyces* strains coupled with ultrasound treatment to accelerate aging on lees, and studied their impacts on the polysaccharide release and on the organoleptic properties of red wine. Cacciola et al. [32] tested the effects of HPUs during wine aging on lees, and found that their effect could be compared with the use of β-glucanases, enzymes which are able to demolish the lees glucans and facilitate the release of intracellular components;
(d) Recover byproducts, such as phenolics from grape pomace [33,34,35] or stilbenes from grape canes [36];
(e) Accelerate the reactions of aging: one other effect of cavitation is the production of highly reactive radical species, such as ^_^OH and ^_^H radicals, that may undergo a range of subsequent reactions, including the generation of H_2_O_2_, and these highly oxidizing species can have a significant effect on both biological and chemical species in aqueous solution [29]. The possible formation of free radicals could help accelerate wine aging reactions [37]. In this way, Zhang et al. [38] have provided the first direct evidence of the formation of the 1-hydroxylethyl free radical (a radical that arises from ethanol oxidation) in red wine exposed to ultrasounds. Lukic et al. [39] has stated that ultrasound treatment might accelerate some aging reactions and shorten the period of wine aging. Zhang and Wang [40] have stated that the ultrasound application did not only temporally influence the color characteristics and phenolic composition of wine, but it also has a longer effect on their evolution during wine storage.

In this work, the interest is focused on the effect that HPUs may have on facilitating the reduction of alcohol content of highly colored wines by the application of those HPUs to crushed grapes with lower sugar content.

## 2. Materials and Methods

### 2.1. Grapes

Monastrell red grapes were all harvested from one vineyard in the province of Murcia (Spain). The vineyard presented two differentiated areas with different maturation stages by a given date. Two separate harvests were done in these two areas, so we had two different batches of grapes from the same vineyard that differed in sugar content. Grapes were transported on the same day to the winery for their processing.

### 2.2. Winemaking (Micro-Vinification)

The less mature grapes (400 kg, 25.4 °Brix, 14 °Baumé) were destemmed and crushed. Half of the crushed grapes were treated (sonicated vinification, US14) with a winery scale power ultrasound system (MiniPerseo, Agrovin S.A., Alcazar de San Juan, Spain) that can treat 400 kg of crushed grapes per hour. The system operated at 2500 W and 28 kHz frequency, with a power density of 8 W/cm^2^. The other half of the crushed grapes was not treated (control vinification, C14). The more mature grapes (200 kg, 29 °Brix, 16 °Baumé) were also destemmed and crushed (control vinification, C16). Small 50 kg, stainless steel tanks (per triplicate) were filled with both control grapes and with the ultrasound-treated crushed grapes. Must homogeneity in each tank was achieved by weighing the solid parts and the liquid must separately, and filling each 50 L vessel with the same quantity and proportion to assure the same solid/liquid ratio in each vessel. Total acidity was corrected, if necessary, to 5.5 g/L, and selected yeasts were added (Viniferm CT007, Agrovin, Alcazar de San Juan, Spain, 20 g of dry yeast/100 kg of grapes). The skin maceration time was 7 days for all the samples. Throughout the fermentation pomace contact period, the cap was punched down twice a day. At the end of this period, the wines were pressed in a 75 L pneumatic press. Free-run and press wines were combined and left at room temperature until the end of alcoholic fermentation. When the fermentation was finished, the wines were racked twice, cold stabilized at 2 °C for one month, and bottled. They were analyzed at the time of bottling. 

### 2.3. Analytical Determinations

The wines were characterized by measuring the alcohol content, pH, total and volatile acidity, and acetic acid, according to European Community methods [41]. Total and reducing sugars, methanol, malic and tartaric acids, ethanal, and gluconic acid were determined by enzymatic methods carried out via an automated analyzer (Miura One, TDI, Barcelona, Spain).

Spectrophotometric parameters: color intensity (CI) was calculated as the sum of absorbance at 620, 520, and 420 nm, and hue as the ratio between absorbance at 420 nm and absorbance at 520 nm. Total and polymeric anthocyanins were determined spectrophotometrically [42]. Total phenol index (TPI) were calculated by measuring wine absorbance at 280 nm, according to Ribereau-Gayon et al. [43]. Total tannins were determined by the methyl cellulose precipitation method [44].

Determination of tannins by the phloroglucinolysis method: Wine samples were prepared as described by Busse-Valverde et al. [45] from an optimization of the method described by Pastor del Rio and Kennedy [46]. In short, 5 mL of wine were evaporated in a centrivap concentrator (Labconco, Kansas City, MO, United States), dissolved in 3 mL of water, and then passed through a C18-SPE column (1 g, Waters, Milford, MA, United States). Compounds of interest were eluted with 10 mL of methanol, evaporated, and then dissolved in 1 mL of methanol. The analyses of tannins were done by depolymerizing the molecule using the phloroglucinol reagent. The depolymerized samples (10 μL injection volume) were analyzed by HPLC [45]. These analyses allowed determination of the total tannin content, the apparent mean degree of polymerization (mDP), and the percentage of each constitutive unit. Wine tannin mass conversion yield was also calculated to be 38.16% ± 5.70%.

### 2.4. Determination of Wine Volatile Compounds by Solid-Phase Microextraction–Gas Chromatography–Mass Spectrometry (SPME-GC-MS)

For the isolation of major volatile compounds by solid-phase microextraction (SPME), a divinylbenzene–carboxen–polydimethylsiloxane (DVB/CAR/PDMS) fiber was used. It was conditioned before the first use by insertion into the gas chromatograph injector, as recommended by the manufacturer. 

For the analysis of wine volatile compounds, 10 mL of wine were added to a 20 mL headspace vial. Four grams of sodium chloride and 50 µL of the internal standard (125 µL/L of 2-octanol in absolute ethanol) were added to the same vial. The vial was sealed and loaded onto a Gerstel auto-sampling device (Gerstel GmbH and Co.KG, Mellinghofen, Germany), and the analysis was conducted using an Agilent 6890N gas chromatograph coupled to an Agilent 7890B single quadrupole mass spectrometer (Agilent Technologies, Santa Clara, CA, United States). The conditions of the microextraction procedure, the gas chromatograph, and the mass spectra conditions can be found in Gómez-Plaza et al. [47]. Peak identification was carried out by comparing mass spectra with those of the mass library (Wiley 6.0), and also by comparing the calculated Kovats retention indices, determined with reference to a homologous standard series of C9–C30 hydrocarbons, with those published in the literature. Semiquantitative data were obtained by calculating the relative peak area (or total ion signal) in relation to that of the internal standard (2-octanol).

### 2.5. Sensory Analysis

Wines were subjected to sensory evaluation using a descriptive test. Prior to the sensory analysis, the wine from the three different replications for each experience was pooled to have a representative sample, and to avoid differences among the replications. Ten staff members with experience in wine sensory analysis and interest in the project were selected for the sensory analysis.

Forty mL of each wine was poured 30 min before evaluation. Glasses were coded and presented to judges in a sensory room that was kept at 20 °C and free of unusual odors. Each panelist sat in an individual isolated booth illuminated with white light. The intensity of each attribute was rated on a scale of zero to five, with a score of zero indicating that the descriptor was not perceived. Data from all the judges for all samples were used in the analysis.

### 2.6. Statistical Analysis

The analysis of variance and the principal component analysis were carried out using the statistical package Statgraphics Centurion XVI.3 (Statpoint Technologies, Inc., The Plains, VA, USA).

## 3. Results

Many of the studies about the characteristics of wines made from grapes with different ripening degrees were done on grapes sampled at different times during ripening. However, it is also well known that, at any given point, the physiological characteristics of grape berries in a vineyard are very heterogeneous [48]. This is especially true in large vineyards, where large changes in vineyard orientation or slope or altitude can be found. Based on that, a separate sampling of two different zones of the same vineyard gave us the opportunity of working with grapes grown under the same conditions but with different ripening stages at harvest time.

Table 1 shows the physico-chemical characteristics of the three studied wines. It is important to point out that the system used in this study differs from those used in previous studies, where HPUs have been applied during wine elaboration, since almost all of them used laboratory scale systems, either ultrasonic baths or probes, whereas in this study a winery scale system with a continuous on-line must treatment has been used.

When comparing C14 with US14, no significant differences were found in any of the physico-chemical properties, except for the content in methanol. Methanol is produced before and during alcoholic fermentation from the hydrolysis of pectins by pectinase enzymes (such as pectin methylesterase), which are naturally present in the fruit. More methanol is produced when must is fermented on grape skins; hence, there is generally more methanol in red wines than in rosé or white wines [49]. The higher degree of degradation that ultrasounds caused in grape skins could have increased the concentration of pectins in the must, and the consequent degradation of these pectins may have increased the concentration of methanol in the US14 wine. However, the concentration was lower than the maximum established by the OIV (400 mg/L). Zhang et al. [50] also found that HPUs did not affect most of the physico-chemical characteristics of wines.

If we compared the C14 and US14 with C16, the differences were as expected. The alcohol content of C16 was almost two units higher than C14 and US14. Differences in residual sugars and pH were small, but total acidity and volatile acidity was higher in C16, with no differences in tartaric acid content (acidity was corrected in the must when necessary). Gluconic acid increased in the wines made from the more mature grapes. Gluconic acid is a product originated by fungi, such as *Botrytis cinereainereal, Penicillium, Aspergillus,* and *Mucor*, and bacteria, such as *Acetobacter* and *Gluconobacter*, and their presence in must and wines is related to the level of grape infection, which is facilitated by climatic factors like moisture and rainfall, as well as by physiological factors like ripening stage [51]. Therefore, detection of gluconic acid allows estimation of the health status of the grape harvest and wine quality within the production cycle at a winery. OIV stated that levels of gluconic acid of 200–300 mg/L or lower indicates sound grapes, whereas levels up to 1.0 g/L indicate an initial stage of fungus infection [52]. The maximum level detected in our wines was 0.34 g/L in C16 wine.

Ethanal was also controlled to determine if the use of HPUs at the beginning of the winemaking process could, somehow, affect the evolution of vinification and the presence of oxidation markers. 

This molecule is produced by ethanol oxidation and although it may, at low concentrations, contribute to red wine color evolution during aging [53], an excessive production of acetaldehyde can result in the appearance of oxidation off-flavor [54]. No differences regarding this compound could be detected between C14 and US14 and C16.

Table 2 shows the chromatic characteristics of the studied wines. When comparing C14 and C16, we can observe a statistically significant increase in color intensity, total phenols, total anthocyanins, polymeric anthocyanins, and total tannins in C16 wine. Previous results from our group [55], studying the physico-chemical and chromatic characteristics of Monastrell grapes harvested at different degree of ripeness, has shown that even when the grapes were harvested at the moment when anthocyanins content was at its maximum, those grapes did not lead to the most intensely colored wines; however, the wines elaborated from grapes harvested three weeks later had better chromatic characteristics and withstood better aging in the bottle. This was due to the fact that the extent of cell wall degradation in overly matured grapes facilitates the extraction of phenolic compounds from skins, coincident with the results found in this study. Also, Perez Magariño and Gonzalez-San José [56] stated that a late harvest led to the highest quality aged wines, due to a more appropriate phenolic composition that led to a higher stability of their color.

When comparing the C14 and US14 wine chromatic parameters, the positive effect of the application of HPUs to the crushed grapes was clearly observed, with the US14 wine presenting significantly higher values for all the chromatic parameters measured than the C14 wine, except for total anthocyanins, which did not differ from C14 wine. Moreover, US14 wine did not statistically differ in any chromatic parameter from C16 wine. Our previous studies in the application of HPUs to crushed grapes have shown the increase in chromatic parameters due to HPUs application and its usefulness for reducing maceration time in wineries [27]. Similarly, Ferraretto and Celotti [57], who also studied the effect of the application of ultrasound to crushed grapes on wine color, demonstrated that the sonicated crushed grapes led to musts and wines with higher polyphenol content, with the extraction of tannins being more favored than that of anthocyanins. El Darra et al. [26] also compared the effect of HPU-treated grapes, using a lab bath, on wine phenolic content, finding an enhancement in the polyphenol content.

Table 3 shows the compositional information of the wine tannins, obtained through a phloroglucinolysis reaction. This methodology not only gives us information on the tannins that can be depolymerized by the phloroglucinol reagent, but also allows us to gain information on the mean degree of polymerization of these tannins and their composition, especially regarding galloylated units and the presence of epigallocatechin subunits. Similarly, to the data observed when tannins were determined by the methyl cellulose method, if we compared both control wines, tannin concentration was higher in C16 wine than in C14 wine. This is related with an easier extraction of these compounds from skins, and, in the same way, the concentration of the subunit epigallocatechin (EGC) was slightly higher in C16 wine, although differences were not statistically significant. EGC is a subunit that only can arise from grape skins, where both prodelphinidins and procyanidins are present, contrary to seeds, where only procyanidins are present. The mean degree of polymerization of C14 and C16 wines was similar whereas a slightly higher percentage of galloylation was observed in the tannins from C16 wine, a parameter that could indicate a larger extraction of seed tannins in C16 wine, probably due to the higher concentration of alcohol in the medium during fermentation favoring the extraction of these tannins.

When comparing C14 and US14 wine, important differences were observed. The concentration of tannins was much higher in the wine elaborated with sonicated grapes. The lower value of mDP, together with the higher value of epicatechin gallate subunit (ECG) in US14 wine indicated a higher extraction of tannins from seeds. Furthermore, the concentration of EGC was similar in C14 and US14 and the most important difference was the concentration of ECG. Up until now, there are no studies where the effect of HPUS on seed integrity has been reported but the results pointed to an effect of HPUS in the easiness of seed tannin extraction. Comparing US14 and C16 wines, US14 wine presented higher tannin concentration and a lower mDP than C16 wine, reiterating the positive effect of HPUS in tannin extraction, which may help to ensure a high wine color stability during storage. 

The application of HPU technology to crushed grapes and its effect on the wine’s volatile compounds has been less studied than its effect on phenolic compounds. Bautista-Ortin et al. [25] studied the application of HPUS to crushed grapes, looking for a reduction on the maceration time needed for the extraction of phenolic and volatile compounds, and they found only small differences in the wine volatile composition. Roman et al. [58] applied ultrasounds to Sauvignon Blanc crushed grapes, and found an increase in thiol compounds, key aroma compounds for Sauvignon Blanc wines. Zhang et al. [59] also studied the effect of ultrasounds on higher alcohol content in wine, reporting a decrease in these compounds, although they applied the HPUs treatment to finished wines to study their evolution during aging and not to crushed grapes.

Table 4 shows how the level of maturity in grapes and the use of HPUs affected the major volatile components of the different wines.

Esters, as one of the most important odorants in wines, provide abundant floral and tropical fruity aromas [60]. The origin of esters in wine is primarily the fermentation process, although they could be present in small amounts in grapes [61]. Two different groups of esters were detected in the wines: the acetates of ethanol and other higher alcohols, and the esters of fatty acid and ethanol. The most abundant esters were isobutyl acetate, isoamyl acetate, ethyl hexanoate, ethyl octanoate, and ethyl decanoate. The majority of them were affected more by grape maturity than by the application of ultrasounds to crushed grapes, which could be expected given the origin of these compounds, and the fact that the ultrasound treatment did not affect the most important must characteristics or the development of the fermentation process (data not shown). The increase in sugar content associated with the more mature grapes, and therefore, the higher production of ethanol and higher alcohols were responsible for the higher concentration of acetates in C16 wine compared with C14 wine, although hexyl acetate was present at higher concentration in C14 wine. Similar results were stated by Zhao et al. [60]. Regarding fatty acid esters, previous results have described a large presence of fatty acids, formed via the lipid metabolism of yeast in the more mature musts [62]; this may favor a higher concentration of their esters in wine. 

The higher presence of both types of esters implies that the sum of esters was higher in C16 wine. These results were also reported by Bindon et al. [63], who found that extended ripening time was associated with increased concentrations of some esters, such as ethyl decanoate and butyl acetate. The sum of total esters was not significantly different when we compared C14 and US14 wine; only small differences in some esters could be observed, with higher concentrations in ethyl decanoate and dodecanoate found in US14. 

The production of higher alcohols is linked to the amino acid metabolism by yeasts, and the alcohol deshydrogenase enzymes in fruit and yeasts are the responsible for catalyzing the reduction of aliphatic aldehydes to alcohols [61]. We found a higher concentration of alcohols in the wine from the more mature grapes, although a decrease in hexanol (leafy, grassy aroma) and heptanol (chemical, green notes) due to higher maturation of the grapes was detected. Benzyl alcohol was not detected in C16 wine, yet 2-phenyl ethanol, with a rose aroma, and octanol were present at higher concentrations in this wine.

Comparing C14 and US14 wine, no difference in the sum of total alcohols could be observed although some differences in individual alcohols were found, such as higher concentrations of 3-methyl-1-butanol, 1-heptanol, or 2-phenylethanol, and lower concentrations of hexanol in C14 wine. Although higher alcohols can contribute to a positive effect on wine aroma if they are not present at very high concentrations [64], Zhang et al. [59] have proposed the use of HPUs to reduce higher alcohol content in finished wines.

Concentrations of linear fatty acids decreased in the wine made with the more mature grapes, except for the concentration of acetic acid, which increased in these wines, as would be expected, because concentrations of sugars also increased. These acids are important in the wine aroma balance, although they may impart an unpleasant fatty odor, and even a rancid smell in wine, when present at high concentrations. Although a higher concentration of fatty acids has been described in must from mature grapes [62], the reduction observed in the wine made from the more mature grapes can be related to the previously observed higher concentration of their corresponding esters. It is clear that a connection between harvest time and concentrations of esters and acids in the wine exists. Studies have shown that the sensory differences observed in Grenache wines made with grapes with different ripening stages could be explained by the variability in the concentration of important major volatile compounds, such as esters and acids [65]. Slightly higher concentrations of fatty acids were measured in C14 wine than in US14 wine; the application of HPUs on crushed grapes led to a modification in these compounds. Restrepo et al. [66] found that anaerobic conditions could favor the accumulation of fatty carboxylic acids, and we hypothesized that, although HPUs may have a degassing effect, the process also implies a higher movement of the crushed grapes and that small amounts of oxygen could be dissolved in the must, justifying the lower amounts of fatty acids in the US14 wine.

Among other compounds, benzaldehyde (almond, burnt sugar notes) can be associated with defective wines [67]. It is probably formed by the oxidation of benzyl alcohol, or by the action of microorganisms on aromatic amino acids [61]. The higher concentration of it in C16 wine may explain the lack of detection of benzyl alcohol in these wines. Terpenes were present at lower concentrations in the wine made from the most mature grapes and in the HPUs-treated grapes, except linalool in C16 wine, where higher concentrations were observed. Curko et al. [68] found that the application of HPUs with the highest amplitude could decrease the quantity of linalool, and García et al. [69] suggested that ultrasounds decreased grape aroma in wine, which could be reasonable since most of aromas come from volatile compounds, and may be easily lost by the degassing effect of ultrasound.

**Figure 1:**
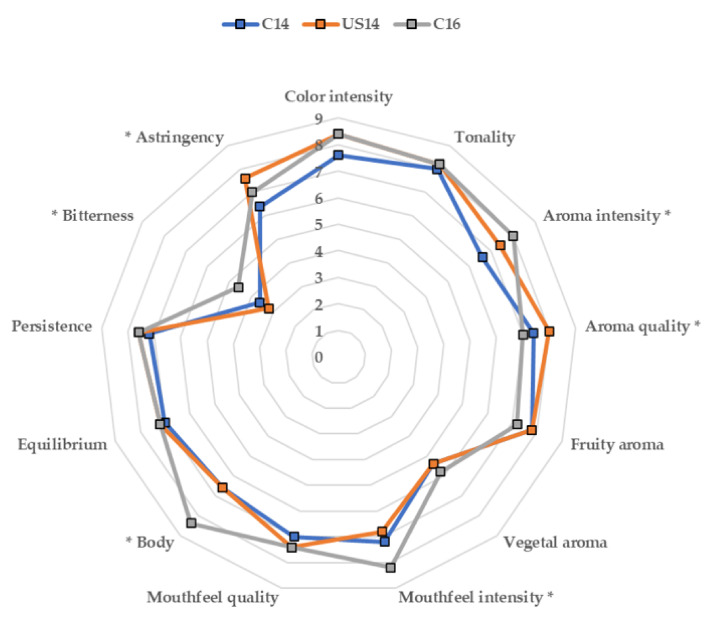
Descriptive sensory analysis of the three different wines (* denotes significant differences *p* < 0.05).

The results up to now point to large differences in the phenolic compounds and chromatic characteristics of wine due to the use of ultrasounds in the winery, while variations in wines’ volatile compounds were not so evident. However, the most important tool researchers have for evaluating if the observed changes in phenolic and volatile compounds due to ultrasounds will have any effect on consumers’ appreciation is the sensory analysis. Wines were subjected to a descriptive sensory analysis, and Figure 1 shows the results of this analysis. It can be seen how with color attributes, color intensity was slightly lower in C14 wine than in C16 and US14 wines, although differences were not significant in either color intensity or tonality among the three wines. On the other hand, significant differences in aroma intensity and quality were observed among wines, with aroma intensity reaching higher scores in C16 wine (probably related to the highest concentration of most of the families of volatile compounds measured in this wine), although aroma quality scored the highest value in US14 wine. Although, as observed previously, C16 wine presented higher concentrations of esters, no differences were observed in fruity aroma, probably because most of these esters could be present at concentrations lower than their odor threshold. Another reason for the higher aroma quality of US14 compared with C16 wine, together with the similarity in fruity aroma perception (although esters were present at higher concentration in C16 wine), can be attributed to the higher alcoholic content of C16 wine. King et al. [14] found that fresh fruit aroma decreased as the alcohol concentration increased, confirming the results of Goldner et al. [13] that ethanol suppresses “fruity” aromas. 

Mouthfeel intensity and body scores were significantly higher in C16 wine (which could be related to its higher alcohol content), and no differences in mouthfeel quality, equilibrium, or persistence could be detected among wines. Bitterness was higher in C16 than in C14 or US14. The greater bitter sensation imparted by C16 wine has to be attributed to the higher alcohol content. Noble [70] reported that bitterness in wine is elicited by flavonoid phenols, which are bitter and astringent, but also by ethanol, and that ethanol enhances wine bitterness intensity and duration. Similarly, Cretin et al. [71] found that the sweetness of dry wines was not affected by ethanol content; however, ethanol had an indirect effect on wine taste by increasing the bitterness perception. Astringency was higher in US14 compared to C14 wine, which is in accordance with the higher concentration of tannins measured in these wines and their higher percentage of galloylation—although that did not differ from the percentage in C16 wine. Previous results showed that delayed-harvest Merlot wines were described as having higher viscosity, sweet taste, and fruit-derived aromas, while early-harvest wines were described by vegetal character, acidity, and low color intensity [64]. In contrast, in Cabernet franc wines, astringency, bitterness, color intensity, and alcohol increased with delayed harvest [72], very similar results to those observed here. The most significative changes that could be attributed to the application of ultrasounds in US14 wine was the higher color and aroma quality and higher astringency.

**Figure 2:**
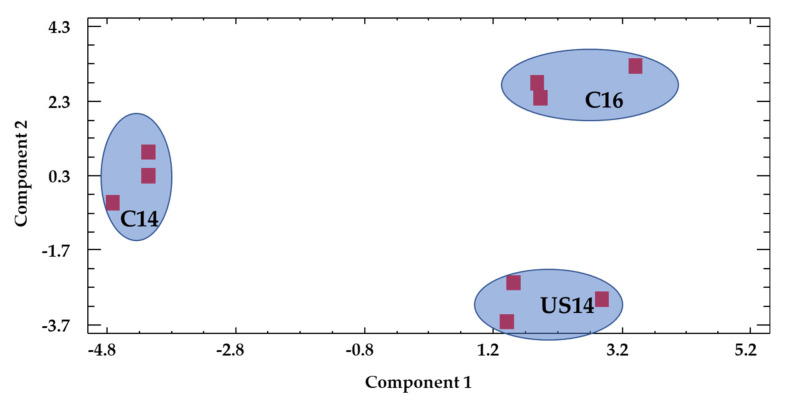
Distribution of the different wine samples in the plane defined by the two first principal components.

**Figure 3:**
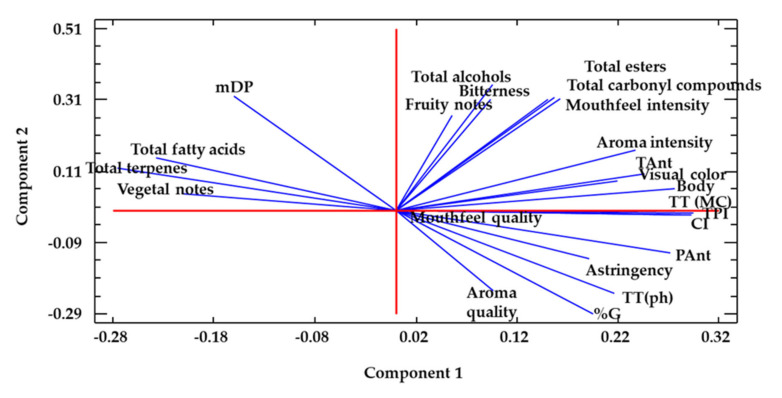
Distribution of the weight of the different variables used in the principal component analysis. CI: color intensity, TPI: total phenol index, TAnt: total anthocyanins, PolAnt: polymeric anthocyanins, TT (MC): total tannins determined by the methylcellulose method, TT(ph): total tannins determined by phloroglucinolysis, %G: percentage of galloylation, mDP: mean degree of polymerization.

A principal component analysis was conducted using all the measured chromatic and phenolic parameters, together with the sum of major aroma compounds and sensory scores as variables. This analysis reduced the information provided by all the measured variables to two principal components, and explained 78% of the variability of the data (Figure 2 and Figure 3). The objective was to find out how the wine samples were grouped, or more exactly, where they were located in the plane defined by the first two principal components, in order to determine how closely the US14 and C16 wine samples were located (which would indicate similarities in their characteristics) and which variables were mainly responsible for the grouping. A clear separation between samples was observed. C14 wine was separated from C16 wine and US14 wine along component 1, while US14 and C16 wines were closely located along component 1, yet clearly separated along component 2.

The different weights of the variables in achieving the sample separation can be seen in Figure 3. Those with the highest loadings in the negative part of component 1, where the C14 wine sample was located, were tannin mDPs, vegetal aroma scores, and two families of volatile compounds (fatty acid content and the concentration of terpenes and norisoprenoids), whereas all the other descriptors were located in the positive part of component 1. US14 wine samples were located in the negative part of component 2, and C16 wine was in the positive part. The descriptors with the highest loadings in the negative part of component 2 were astringency, total tannins (determined by phloroglucinolysis), percentage of galloylation, and the aroma quality, which is quite coincident with the observed results of the analytical variables. This analysis clearly indicates that the use of HPUs induces a modification in the wine composition, especially the chromatic composition and sensory characteristics, leading to a wine that shares more characteristics with the one made with the more mature grapes, while maintaining a lower alcohol content.

## 4. Conclusions

In conclusion, the results showed that the wine obtained with ultrasound-treated grapes presented chromatic characteristics that did not differ to those of a wine obtained from more mature grapes, and reached the highest scores in aroma and mouthfeel quality descriptors in a sensory analysis. The use of ultrasound technology, being a clean, eco-friendly, and energetically very efficient technology—and not less importantly, being an authorized practice in wineries—could be an interesting option for obtaining wines with similar color intensity and sensorial quality parameters as wines obtained from more mature grapes, but with a lower alcohol content.

## Figures and Tables

**Table 1:** Physico-chemical characteristics of the studied wines.

	%Alc	RS	TS	pH	Tac	Vac	MeOH	Mal	Tart	Ethanal	Gluc
C14	14.7 ± 0.2a	1.8 ± 0.1a	2.4 ± 0.1a	3.7 ± 0.02b	5.0 ± 0.1a	0.6 ± 0.01a	192.7 ± 2.5a	0.8 ± 0.1b	1.4 ± 0.1a	55.3 ± 3.1a	0.09 ± 0.01a
US14	14.6 ± 0.46a	2.1 ± 0.1a	2.6 ± 0.1a	3.7 ± 0.01b	5.1 ± 0.10a	0.6 ± 0.01a	261.0 ± 9.5c	0.8 ± 0.1b	1.5 ± 0.1a	55.3 ± 9.0a	0.10 ± 0.01a
C16	16.217 ± 0.1b	3.4 ± 0.1b	4.1 ± 0.b	3.6 ± 0.01a	5.7 ± 0.1b	0.9 ± 0.01b	217.7 ± 9.7b	0.5 ± 0.1a	1.4 ± 0.1a	52.7 ± 3.5a	0.34 ± 0.04b

%Alc: alcohol content, RS: reducing sugars (g/L), TS: total sugars (g/L), Tac: Total acidity (g/L), Vac: volatile acidity (g/L), MeOH: methanol content (mg/L), Mal: malic acid (g/L), Tart: Tartaric acid (g/L), ethanal (mg/L), Gluc: Gluconic acid (g/L). Different letters within the same column indicate significant differences (*p* < 0.05).

**Table 2:** Chromatic and phenolic characteristics of the studied wines.

Sample	CI	Hue	TPI	TAnt	PolAnt	TT (MC)
C14	14.34 ± 0.49a	0.54 ± 0.01b	47.95 ± 1.12a	407.63 ± 17.70a	73.07 ± 5.18a	1444.13 ± 35.36a
US14	17.84 ± 1.22b	0.56 ± 0.01c	60.46 ± 3.56b	453.23 ± 39.42ab	98.95 ± 5.13b	1930.31 ± 27.42b
C16	17.97 ± 0.79b	0.53 ± 0.01a	60.57 ± 1.56b	475.49 ± 19.65b	92.67 ± 3.83b	1972.49 ± 47.60b

CI: color intensity, TPI: total phenol index, TAnt: total anthocyanins (mg/L), PolAnt: polymeric anthocyanins (mg/L), TT (MC): total tannins (determined by the methylcellulose method, mg/L). Different letters within the same column indicate significant differences (*p* < 0.05).

**Table 3:** Concentration and composition of total tannins determined by the phloroglucinolysis method.

	TT (mg/L)	mDP	%Gal	EGC (µM)	ECG (µM)
C14	698.22 ± 21.14a	6.21 ± 0.02c	2.74 ± 0.01a	398.11 ± 27.84a	62.22 ± 11.45a
US14	951.47 ± 46.98c	4.91 ± 0.07a	4.16 ± 0.01c	394.25 ± 31.66a	129.71 ± 17.82b
C16	801.44 ± 17.12b	6.03 ± 0.11b	3.12 ± 0.02b	451.39 ± 25.51a	71.36 ± 9.53a

TT: total tannins, mDP: mean degree of polymerization, %Gal: percentage of galloylation, ECG: concentration of epicatechin gallate subunit, EGC: concentration of epigallocatechin subunit. Different letters within the same column indicate significant differences (*p* < 0.05).

**Table 4:** Major volatile compounds in the control wines and in the wine from ultrasound-treated grapes (µg of 2-octanol equivalents/L).

	Control 14	US14	Control 16
*Esters*			
2-Methylpropyl acetate	1290a	1246a	2140b
Ethyl butanoate	7a	9a	15b
Ethyl 2-methylbutanoate	10b	2a	3b
Ethyl 3-methylbutanoate	4b	2a	4b
3-Methylbutanol acetate	316a	252a	377b
Ethyl hydrogen succinate	2a	3a	5a
Ethyl hexanoate	440a	432a	392a
Hexyl acetate	89b	8a	4a
3-Hexenyl acetate	0a	1b	0a
Ethyl heptanoate	10a	13a	28b
Ethyl 2-hexenoate	15a	13a	12a
Methyl octanoate	10a	13a	11a
Ethyl octanoate	1760a	1672a	2049b
Isopentyl hexanoate	17a	23b	31c
Ethyl decanoate	1012a	1343b	1422b
3-Methylbutanol octanoate	36a	38a	46a
Diethyl succinate	36a	32a	44b
Ethyl 9-decenoate	30b	14a	19a
Ethyl undecanoate	25b	13a	20b
2-Phenylethyl acetate	115b	80a	118b
Ethyl dodecanoate	144a	251b	244b
Pentyl decanoate	25a	28a	32a
Sum esters	5445a	54657a	7184b
*Alcohols*			
Propanol	0a	2b	2b
2-Methylpropanol	146b	115a	161b
Butanol	1a	4b	8c
3-Methylbutanol	2181a	2048a	3066b
4-Methylpentanol	1a	1a	3b
3-Methylpentanol	4a	3a	6b
Hexanol	95b	119c	75a
Heptanol	22c	82a	13b
2-Ethylhexanol	8a	27c	13b
Octanol	15a	17a	21b
2,3-Butanediol	26a	22a	42.22b
Methyl thiopropanol	18a	12a	2a
Benzyl alcohol	26b	0a	0a
2-Phenylethanol	1849b	1457a	2324c
Sum alcohols	4393a	3836a	5734b
*Carbonyl compounds and lactones*			
2-Octanone	8a	11b	8a
Benzaldehyde	13a	16a	20b
3-Hydroxy-2-butanone	84a	82a	169b
γ-Butyrolactone	6b	0a	12c
Sum ketones	111a	110a	209b
*Acids*			
Acetic acid	106a	81a	164b
Propanoic acid	7b	14b	0a
Butanoic acid	34a	10a	30a
Hexanoic acid	73b	27a	21a
Octanoic acid	161b	72a	43a
Sum acids	380b	205a	258a
*Terpens and norisoprenoids*			
Limonene	6a	4a	4a
Terpinolene	9a	6a	3a
(+)-cis-m-Menth-8-en	18b	14b	0a
β-Ionone	65b	12a	20a
Linalool	17a	16a	40b
Terpinen-4-ol	3b	0a	0a
Citronellol	71b	27a	17a
Sum	189b	80a	83a

Different letters within the same row indicate significant differences (*p* < 0.05).
